# A face detection bias for horizontal orientations develops in middle childhood

**DOI:** 10.3389/fpsyg.2015.00772

**Published:** 2015-06-08

**Authors:** Benjamin J. Balas, Jamie Schmidt, Alyson Saville

**Affiliations:** Department of Psychology, Center for Visual and Cognitive Neuroscience, North Dakota State UniversityFargo, ND, USA

**Keywords:** face detection, spatial vision, visual development

## Abstract

Faces are complex stimuli that can be described via intuitive facial features like the eyes, nose, and mouth, “configural” features like the distances between facial landmarks, and features that correspond to computations performed in the early visual system (e.g., oriented edges). With regard to this latter category of descriptors, adult face recognition relies disproportionately on information in specific spatial frequency and orientation bands: many recognition tasks are performed more accurately when adults have access to mid-range spatial frequencies (8–16 cycles/face) and horizontal orientations (Dakin and Watt, 2009). In the current study, we examined how this information bias develops in middle childhood. We recruited children between the ages of 5–10 years-old to participate in a simple categorization task that required them to label images according to whether they depicted a face or a house. Critically, children were presented with face and house images comprised either of primarily horizontal orientation energy, primarily vertical orientation energy, or both horizontal and vertical orientation energy. We predicted that any bias favoring horizontal information over vertical should be more evident in faces than in houses, and also that older children would be more likely to show such a bias than younger children. We designed our categorization task to be sufficiently easy that children would perform at near-ceiling accuracy levels, but with variation in response times that would reflect how they rely on different orientations as a function of age and object category. We found that horizontal bias for face detection (but not house detection) correlated significantly with age, suggesting an emergent category-specific bias for horizontal orientation energy that develops during middle childhood. These results thus suggest that the tuning of high-level recognition to specific low-level visual features takes place over several years of visual development.

## Introduction

A fundamental question in visual development is how infants and children process faces as they develop experience and expertise with facial images. We know, for example, that biased experience with face categories defined by age (Cassia et al., 2009), race (Kelly et al., 2007), and species (Pascalis et al., 2002), leads to subsequent differences in how well faces belonging to these categories can be discriminated from one another in infancy (Scott et al., 2007; Kelly et al., 2009). Biased experience can continue to impact face processing into early childhood as well. Changes in the statistics of face exposure may still lead to changes in recognition performance for children who are 5–6 years-old, for example, such that children whose face environment changes during childhood can undergo a ‘flip’ in the well-known other-race effect. Specifically, Sangrigoli et al. (2005) demonstrating that Korean children adopted by French families between ages 3 and 9 eventually developed an ‘other-race’ effect for Korean faces, despite their prior biased experience with Korean faces, suggesting that there is sufficient plasticity at these ages for face-specific visual learning. Children at these younger ages also undergo improvement in their face recognition abilities brought on by the onset of school (De Heering et al., 2014), suggesting that continuing experience with faces during this age span has direct impact on how faces are processed.

Besides changes in what recognition tasks children are able to accomplish as a function of development and expertise, there is also evidence that *how* children recognize faces changes during early and middle childhood as well. In particular, there are a number of results suggesting that the visual features children use to recognize, discriminate, and detect faces change during childhood. We will call the selective use of a subset of visual features to support face recognition an example of an *information bias*, and we suggest that understanding the nature of these biases and their developmental trajectory offers important insights into how visual recognition develops and how maturation and experience shape high-level processing in quantitative terms based on low-level features. Children’s developing representations of facial appearance for detection and recognition have, however, largely been examined using qualitative descriptions of face processing and we suggest that complementing this body of work with studies examining the way low-level features contribute to recognition during development is an important goal. There are several examples of qualitative or higher-level visual features that have been the target of developmental research into how information is used for recognition. For example, since adult face recognition appears to exhibit category-specific properties that are consistent with ‘configural’ or ‘holistic’ processing (Maurer et al., 2002), the development of face recognition in childhood has often been described vis-à-vis the behavioral indices of these putative mechanisms for face recognition. For example, the extent to which children exhibit the classic composite face effect (Young et al., 1987) has been examined as one indicator of when mature, adult-like face processing has been achieved (De Heering et al., 2007; Mondloch et al., 2007). Similarly, children’s sensitivity to changes in the arrangement of parts with a face (often referred to as the ‘second-order configuration’ of a face) relative to their sensitivity to changes in the local shape of these same parts (often referred to as ‘featural processing’) has also been used to characterize the development of face recognition during childhood (Freire and Lee, 2001; Mondloch et al., 2004). While these results are important and offer a useful description of how category-specific mechanisms for face recognition change during childhood, one weakness of these studies is that it is not clear what visual information supports the behavioral effects used as proxies for adult-like face processing. What exactly leads to holistic processing as measured in the composite face effect or the part-whole task (Tanaka and Farah, 1993)? While there have been attempts to model these effects using low-level features, we have yet to achieve a deep understanding of how to account for these effects in terms of specific visual features. In some cases, like second-order face geometry, we actually know that it is highly unlikely that observers use these specific measurements (e.g., interpupillary distance) for face recognition and detection since these features are not available or diagnostic in natural scenes (Gaspar et al., 2007; Taschereau-Dumouchel et al., 2010). Characterizing development using these kinds of intuitive feature vocabularies is thus potentially less relatable to computational and neural mechanisms, since we as yet lack a clear understanding of how to describe these kinds of features in quantitative terms.

However, face recognition and detection also depend on specific low-level and mid-level visual features. Converging results from computational analyses of face images and behavioral experiments carried out with adult observers demonstrate that the human visual system makes use of particular sub-sets of visual information for face recognition tasks, often in a manner that reflects the diagnosticity of visual features for image classification. Specifically, we will focus here on low-level features that relate easily to properties of the early visual system. While the response properties of neurons in early visual cortex may not be easy to relate to our subjective experience of face images as being comprised of face parts like the eyes, nose, and mouth, we argue that examining how face recognition depends on the feature vocabulary implemented in early vision provides important insights into how neural processing proceeds from low-level measurements to high-level judgments. Also, considering face recognition in terms of these features, rather than qualitative descriptors of more complex face properties like the shape of the eyes or mouth, makes it straightforward to develop a quantitative description of how specific features contribute to recognition. For example, face recognition relies on mid-range spatial frequencies, typically defined as those between 8 and 16 cycles per face (Costen et al., 1994, 1996; Näsänen, 1999; Ruiz-Soler and Beltran, 2006). Faces lacking spatial frequency content in this band are typically more difficult to recognize (Gold et al., 1999) whether they are upright or inverted (Gaspar et al., 2008), with some variation depending on the particular task observers are asked to complete (Goren and Wilson, 2006).

Besides specific spatial frequency bands, adult observers also use specific orientation sub-bands for face recognition: horizontal orientation energy appears to be more useful than vertical orientation energy (Dakin and Watt, 2009). Faces that have been filtered so that primarily horizontal orientation energy is included are recognized more easily than faces filtered to exclude horizontal orientations and are also classified according to emotion more easily (Huynh and Balas, 2014). Sensitivity to the information carried by horizontal orientations also correlates significantly with overall face recognition ability, which suggests a direct functional link between the information in this orientation sub-band and face recognition aptitude (Pachai et al., 2013). Moreover, horizontal orientation sub-bands also appear to carry category-specific behavioral effects like face adaptation and the well-known face inversion effect (Goffaux and Dakin, 2010) and interact with spatial frequency content such that joint tuning for optimal spatial frequency and orientation sub-bands is evident in face processing (Goffaux et al., 2011). Human observers’ reliance on horizontal orientations for face processing is also in accord with computational results demonstrating that horizontally oriented features consistent with the so-called “biological bar-code” for face processing (Viola and Jones, 2001) are among the most diagnostic features for detecting faces in natural scenes. Face processing thus depends in part on critical bands of spatial frequency and orientation information that are potentially extracted from natural images at the earliest cortical stages of visual processing. Understanding both the nature of these biases for information and the subsequent impact of these biases on downstream processing is an important means of linking early vision to high-level judgments.

Over what developmental time frame do biases for particular spatial frequencies and orientations emerge? Unlike holistic or configural processing, these measurements are easily described quantitatively and directly map onto specific neural systems, making them a potentially vital means of describing the development of information biases in the context of specific computational and neural processes. In the present experiment, we therefore chose to investigate the emergence of a bias for horizontal orientations in face processing during middle childhood (5–10 years of age). Compared to spatial frequency, orientation biases for object recognition have only recently been examined in depth, and to our knowledge there is little developmental data describing how infants or children process faces as a function of orientation content. Middle childhood is an important target for research into the ‘tuning’ of high-level vision for specific low or mid-level features, since there is evidence that important changes in face recognition are taking place during this span of development (though see Crookes and McKone, 2009 for an important discussion of how to establish that such effects are face-specific). For example, children 4–6 years of age appear to weigh 2D face pigmentation and 3D face shape differently than adults when judging own- and other-race face similarity (Balas et al., 2015). While the use of high vs. low spatial frequencies appears to be relatively established by age 5 (Deruelle and Fagot, 2005), some aspects of face-specific tuning for mid-band frequencies do not appear to be mature until 9–10 years of age (Leonard et al., 2010). Consistent with the possible slow development of various forms of perceptual tuning to information in face images, children between 4 and 10 also appear to increasingly exhibit a “left-side bias” for face recognition that is species-specific (Balas and Moulson, 2011) and also gradually acquire a bias favoring internal features of the face for recognition over the external contour (Campbell et al., 1995). Considered together, these results suggest that face representations change during middle childhood, converging on an efficient vocabulary for describing face structure that will likely also exhibit information biases for low-level features that are evident in adulthood.

We therefore hypothesized that preferential use of horizontal orientation energy may increase during middle childhood, reflecting a gradual tuning of face processing mechanisms to diagnostic low- and mid-level structures in face images. To test our hypothesis, we presented children between the ages of 5–10 years with a simple object detection paradigm in which they were asked to rapidly categorize images as either faces or houses using a touchscreen interface. To examine the use of specific orientation sub-bands for object detection, we filtered target images to either include primarily horizontal orientations, primarily vertical orientations, or a combination of both orientation sub-bands. We predicted that a bias favoring horizontal orientation energy may increase over our target age range, but only for face images. We expected that observers’ use of specific orientation sub-bands for house detection would either remain stable over the target age range, or potentially rely on different combinations of orientation energy than face images. Our main results supported our initial hypothesis, suggesting that children do become tuned to diagnostic low-level features during middle childhood and that this tuning is category-specific and not simply a consequence of global cognitive and perceptual development (Crookes and McKone, 2009; Williams et al., 2009).

## Materials and Methods

### Subjects

We recruited a total of 43 child participants between the ages of 5–10 years-old to take part in this study (see**Table 1** for a breakdown of participant age). All participants, per parental report, were free of visual or neurological impairments. Written informed consent was obtained from parents or legal guardians for each participant and children 7 years of age or older also provided written assent. All experimental protocols, including procedures for obtaining informed consent, were approved by the North Dakota State University IRB.

**Table 1:** Average response time (calculated from ex-Gaussian parameter values) as a function of age and stimulus condition.

Age (years)	*N*	Face-H (seconds)	Face-V (seconds)	Face-HV (seconds)	House-H (seconds)	House-V (seconds)	House-HV (seconds)
5	6	2.44	2.47	2.26	2.61	2.52	2.56
6	5	2.54	2.54	2.57	2.52	2.66	2.71
7	9	2.19	2.26	2.27	2.37	2.41	2.27
8	7	2.56	2.51	2.41	2.78	2.71	2.66
9	9	1.96	2.07	2.04	2.19	2.11	2.09
10	6	1.69	1.84	1.67	1.82	1.98	1.73

### Stimuli

**FIGURE 1:**
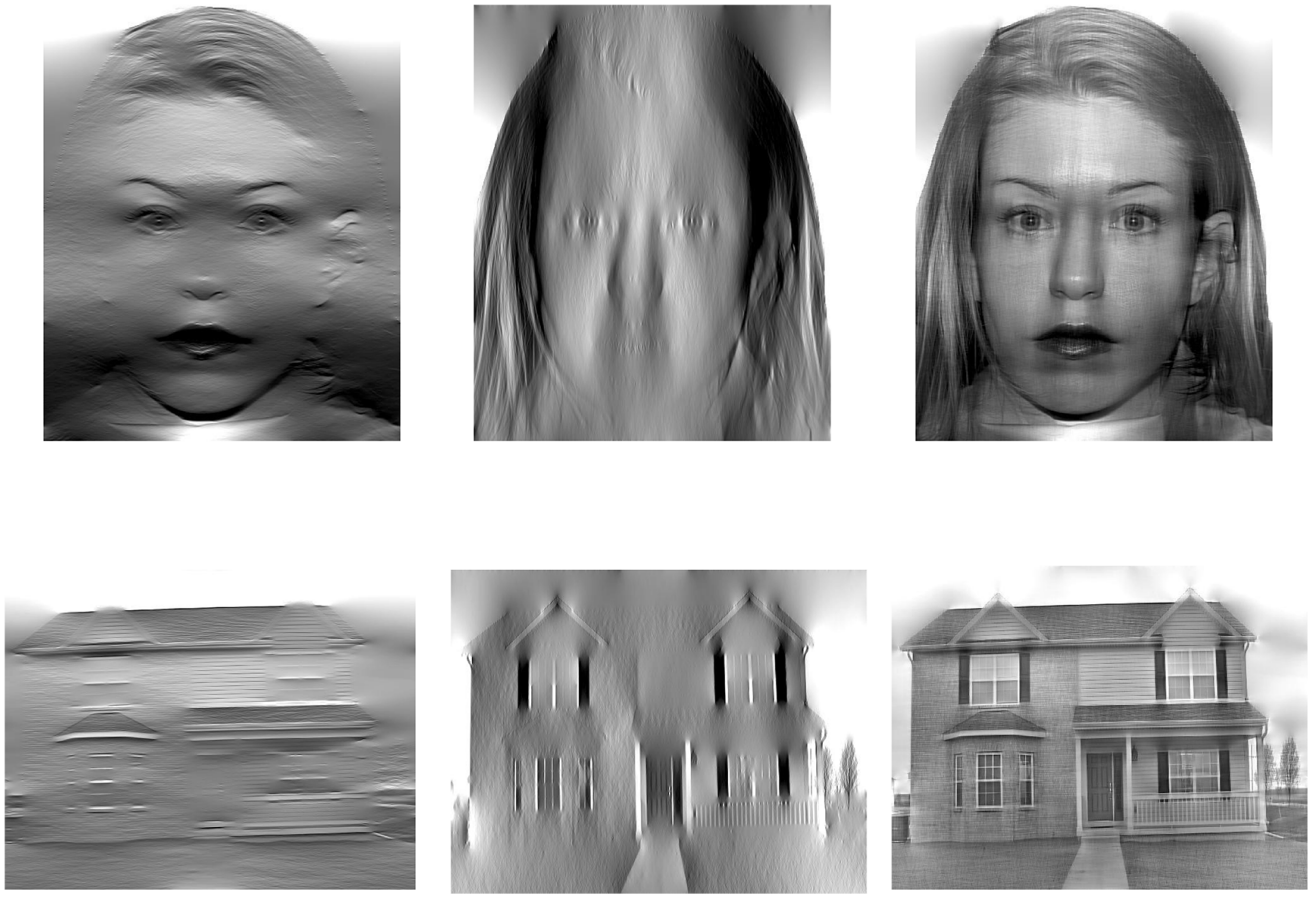
**Examples of faces and houses with bandpass orientation filters applied to limit the orientation energy to horizontal and vertical bands, as well as a combination of both orientation passbands**.

Our stimulus set was comprised of grayscale images of faces (Tottenham et al., 2009) and houses that were filtered so that they contained primarily horizontal orientation energy, primarily vertical orientation energy, or orientation energy from both horizontal and vertical pass-bands. Specifically, following a Fourier transform, the power spectrum of each original image was windowed with a Gaussian envelope centered at the target orientation (0 or 90#x000B0;) with a standard deviation of ∼14#x000B0;. The stimuli were then reconstructed by carrying out an inverse Fourier transform with the transformed power spectrum and the original phase information. This manipulation limits the orientation energy present in the image to orientation sub-bands within the windowing function, yielding a stimulus image that is dominated by orientations close to the target angle. To create images with both horizontal and vertical sub-bands included, the windowing function included both the horizontal and vertical windows, allowing orientation energy from both sub-bands to pass through (**Figure 1**). House images depicted a frontal view of a single house, while face images depicted a frontally viewed face with a neutral expression. The original images in both categories were resized to 400 × 400 pixels following orientation filtering and we matched the mean luminance and global image contrast across all images. We note that these latter manipulation do not guarantee that all low-level properties of the stimulus images are matched (local contrast may vary across images and categories, for example, when pixel intensity values are matched), but nonetheless ensure that some global image properties are closely controlled.

### Procedure

**FIGURE 2:**
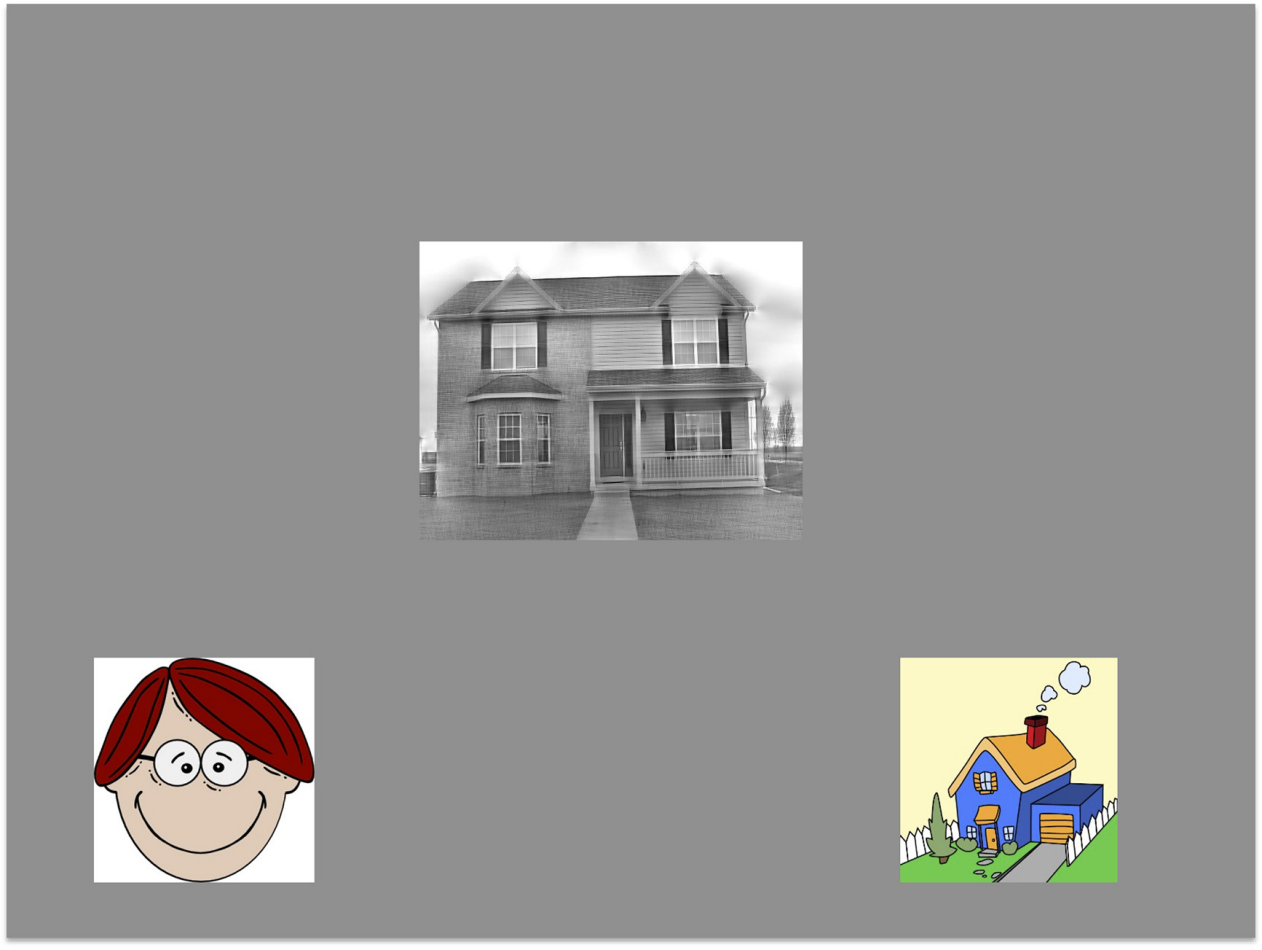
**An example trial from our task: participants were asked to categorize the central image by touching the cartoon image at the bottom of the screen that was of the same object category**.

Participants viewed the stimuli on an 800 × 600 Elo touchscreen display. On each trial, a single image was presented in the center of the display. This image was either a face or a house, filtered either to contain only horizontal orientation energy, only vertical energy, or both orientations. Object category and orientation condition were pseudo-randomized for each participant. Accompanying the central image were two cartoon images of a house and a face that appeared at the bottom of the display offset to the left and right. These images were used as on-screen response buttons for participants to use during the task: to categorize the central image as a face, participants were asked to touch the cartoon face as quickly as possible, and similarly to touch the cartoon house if the central image depicted a house (**Figure 2**). The left/right position of the cartoon images was pseudorandomized across trials so that participants were required to actively attend to the position of the response items as well as successfully classify the central image. Participants viewed the images from an approximate distance of 30–40 cm, though viewing distance varied according to what was a comfortable reaching distance for participants. Head position and eye movements were not fixed or recorded during testing.

Participants completed 24 trials per condition for a grand total of 144 trials in the entire task. Most participants completed the testing session in 10–15 min. All stimulus display and response collection routines were carried out by custom software written using the Matlab Psychophysics toolbox (Brainard, 1997; Pelli, 1997).

## Results

### Accuracy

For each participant, we computed how frequently they chose the correct object category for face and house targets in each orientation condition. In general, accuracy was very high across all ages, averaging over 90% in all conditions. We did, however, remove one 5-years-old participant from our subsequent RT analysis on the basis of poor accuracy (at or below 50% in all conditions) which suggested a failure to comply with task instructions. Our final sample for response time analysis was thus comprised of 42 children. Aside from this, given the overall very high performance observed in all age groups, etc. we did not conduct any further analysis of the accuracy data. We designed our detection task such that we expected participants to perform at ceiling with respect to accuracy (which they did) and to subsequently look for effects of object category and stimulus appearance on response latency.

### Response Times

To determine the extent to which participants’ exhibited a category-specific bias for horizontal orientations that emerged during middle childhood, we examined response latencies to correctly labeled faces and houses. We chose to model child response time distributions using an ex-Gaussian distribution (1) fit to the data obtained in each condition. While a ‘trimmed mean’ (calculated by computing the mean after removing latencies that fall outside some pretermined range) or a median RT are frequently used to describe average response latency, modeling response time distributions has many important advantages (Whelan, 2008). The median is known to be a biased measure of central tendency (Miller, 1988), for example, providing an overestimate of population medians. Also, both the robustness of trimmed mean and median to outlying values means that long RTs cannot contribute to these measures of central tendency, despite the fact that they may carry useful information about performance. For example, Palmer et al. (2011) examined visual search RTs using several candidate distributions (including the ex-Gaussian) and concluded that distributions with an exponential component tended to fit the data more closely than those that lacked one. They concluded that the oft-ignored “junk” data comprised of long response latencies should likely be included in RT analyses. We thus focus on modeling our own data within this framework. The ex-Gaussian distribution is known to be a good fit to behavioral response latencies (Lacouture and Cousineau, 2008) and is particularly useful for coping with data that likely contains outlying data points (which is frequently the case in child response time data). The ex-Gaussian distribution is described by three parameters: mu and sigma, which correspond to the mean and standard deviation of the Gaussian component of the distribution, and tau, which corresponds to the exponential component. Though the mu and tau parameters have been linked to specific cognitive mechanisms (namely sensory and decision-making processes, respectively), presently we chose simply to use these parameters to obtain an estimate of the distribution mean, which we can compute by adding these two parameters together.

$$f(x|\text{μ,σ,τ})=\frac{1}{\tau }\exp\left[\frac{\text{μ}}{\text{τ}}+\frac{{\text{σ}}^{2}}{2{\text{τ}}^{2}}-\frac{x}{\text{τ}}\right]\Phi \left[\frac{x-\text{μ}-{\text{σ}}^{2}/\text{τ}}{\text{σ}}\right](1)$$

Having computed the mean response latency per condition using the ex-Gaussian distribution, we continued by computing a composite score designed to reflect the extent to which participants’ exhibited a bias favoring horizontal orientations over vertical orientations for face and house detection. Since prior results suggest that horizontal orientation energy is favored for face recognition, we predicted that participants might be faster to detect horizontally filtered faces than their vertically filtered counterparts. As such, we computed the horizontal bias index for each object category (face and house) as follows:

$$HBias=\frac{RT_{horizontal}-RT_{vertical}}{RT_{horizontal+vertical}}(2)$$

This index should be more negative when observers are slower to respond to vertically filtered images than horizontally filtered images, and more positive when the opposite is true. Also, the difference in speed between horizontally- and vertically-filtered images is normalized by the response latency when both orientations are included in the image so that baseline differences in speed are used to scale the difference between the critical conditions. To provide an overview of the absolute RTs observed as a function of age and stimulus condition, **Table 1** contains the mean RT for each age group in each condition.

**FIGURE 3:**
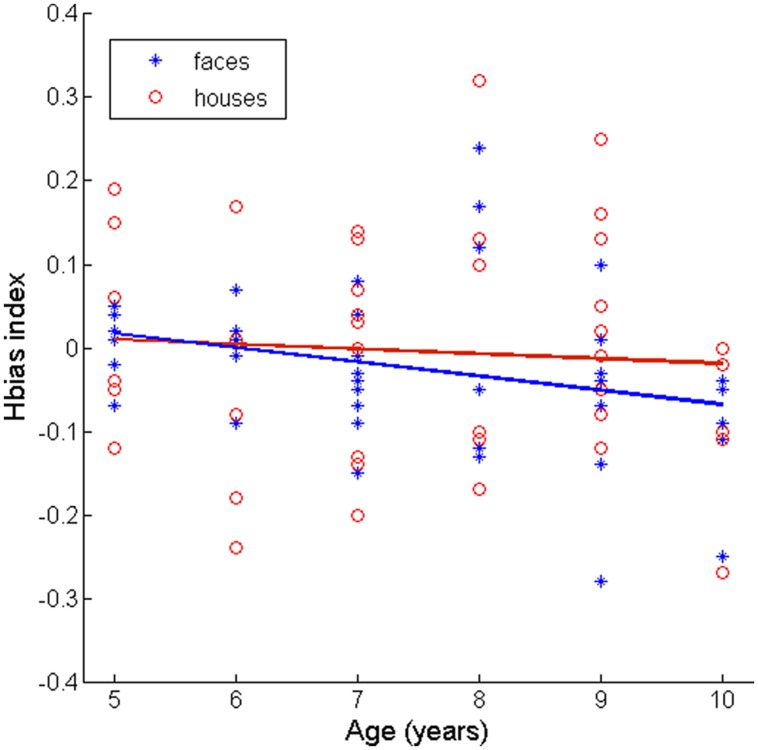
**Scatterplots depicting the value of the Horizontal bias index described in the text and age (binned here by years of age) for face and house targets**. The overlaid lines depict the least-squares fit of each age vs. bias index relationship. We note that some values within age bins may be very close to one another and thus be difficult to see as separate in this plot.

To examine the relationship between participant age and horizontal bias in face and house detection, we tested for significant positive correlations between age and the horizontal bias index described above. We found that age and the horizontal bias index were significantly correlated for faces (Spearman’s ρ = 0.32, *p* = 0.017, one-tailed test, see **Figure 3** for scatterplot) but that this correlation did not reach significance for houses (Spearman’s ρ = 0.096, *p* = 0.27, one-tailed test, see **Figure 3** for scatterplot).

These results thus suggest that older children have a larger bias than younger children for horizontal orientation energy during face detection, but that this is not the case for house detection. This result is consistent with our initial hypothesis that children may develop an information bias favoring horizontal orientation energy during middle childhood, and moreover, that this bias may exhibit category-selectivity. We conclude, therefore, that children are continuing to optimize their representation of faces in terms of low-level features during this developmental period. We continue by discussing this result in the context of other developmental and computational descriptions of visual learning.

## Discussion

We observed that children’s information bias favoring horizontal orientation energy for face detection increases with age during middle childhood. The ‘tuning’ of face recognition to diagnostic low-level features measured by early vision thus appears to be a relatively slow developmental process that may reflect either the statistics of the visual environment, or a maturational process that increases the efficiency of high-level recognition by streamlining the low-level features that contribute to subsequent recognition tasks. At present, we do not attempt to distinguish between these possibilities, offering instead the result that the bias for horizontal orientation energy does change over this age range and also that the bias appears to be category-specific, since there is not observable change in how horizontal and vertical orientation sub-bands contribute to house detection in our task. This result is compatible with a number of other examinations of specific low-level and mid-level information biases that also appear to develop over middle childhood, many examples of which we described in the introduction. We suggest that a key challenge for future work that aims to describe how high-level recognition is supported by low-level and mid-level features is to identify the relationships between these various candidate features (if any) so we can begin to identify core computational mechanisms of recognition. In particular, it would be very useful to examine both the spacing manipulations used to study configural processing in child observers and the manipulations of appearance used to study holistic processing within the context of low-level spatial frequency and orientation features. To the extent that we can establish a link between these well-established but poorly characterized properties of face recognition and specific computations that account for the observed data, the development of visual recognition could be described via a synthesis of behavioral, computational, and neural results. While there have been some attempts to link SF to holistic processing in adults (Goffaux and Rossion, 2006) and also to examine how the face inversion effect manifests as a function of orientation content (Goffaux and Dakin, 2010), to our knowledge there have been few such studies with a developmental focus on our target age range. Of additional importance would be an examination of how the development of invariant recognition (the ability to cope with identity-preserving changes of appearance like viewpoint and expression variation) is or is not tied to specific low-level features like these.

With regard to neural data, it would also be very useful to examine the impact of low-level features on children’s developing neural responses to face and non-face images. Adult observers exhibit clear effects of information content on face-sensitive neural responses like the N170 (Bentin et al., 1996), with orientation energy modulating neural responses in a manner that is consistent with behavioral data (Jacques et al., 2014). Children’s ERP responses also exhibit category-specificity with regard to low-level appearance manipulations that remove critical features that can be used for recognition. Inversion and contrast negation both affect children’s N170 response to faces, for example (Itier and Taylor, 2004). Moreover, the impact of contrast negation on N170 responses to human and monkey faces is species-specific in young children (Balas and Stevenson, 2014), which demonstrates that children’s use of either specific phase relationships or pigmentation cues to recognize human faces is reflected in their neural response to those images. Generally, the N170 is known to change during childhood in terms of its latency and the presence of the frontal P170 component (Taylor et al., 1999), which may reflect very broad developmental changes in the representations and processes supporting high-level vision. While the N170 component exhibits these broad changes in response properties during childhood, its selectivity for faces vs. objects appears to be stable between the ages of 4–10 years of age (Kuefner et al., 2010) implying that category-specific representations are established neurally, but may not yet be tuned to low-level features in an adult-like way. Examining the N170 component in terms of category-specific information biases should provide a detailed account of how children tune cortical representations of appearance for efficient face recognition and detection.

Finally, we note that both behavioral and neural studies of children’s developing representations of face appearance for recognition would be well-informed by continued exploration of other candidate feature vocabularies that have either an explicit computational or neural basis (or preferably both). In particular, elaborating the representation of facial appearance between low-level and high-level vision will eventually require that we examine candidate feature vocabularies that may be implemented at intermediate stages of the ventral visual stream. Identifying such representations is a hard problem that is being studied across multiple disciplines, but we suggest that there are already several examples of useful features that have yet to be examined in a developmental context. For example, adult observers appear to make use of diagnostic micropatterns in face images that are of ‘intermediate complexity’ (Ullman et al., 2002). These features can be computationally derived by searching for image fragments that carry the most information (in an information-theoretic sense) about object category. Fragments that are information-rich are useful for computational face detection, support faster categorization in adults, and also appear to drive face-sensitive ERP and fMRI responses (Harel et al., 2007, 2011; Lerner et al., 2008). Examining how features like these emerge developmentally may be an important indicator of how intermediate stages of face processing change over time. Besides these “fragments” that carry useful information for face recognition, there are also more general-purpose features like SIFT operators or HOG (histogram of oriented Gaussians) features that are designed to support machine vision applications, but may nonetheless offer insights into human vision as well. Advances in visualizing the representation of arbitrary images and objects achieved using such features (Vondrick et al., 2013) may provide important methodological tools for studying these representations developmentally and developing a comprehensive account of the development of face recognition. Overall, a continued emphasis on linking behavior to specific computations and visual representations of complex images will lead to broad advances in understanding how face and object recognition develop, and what mechanisms support efficient processing over developmental time.

## Conflict of Interest Statement

The authors declare that the research was conducted in the absence of any commercial or financial relationships that could be construed as a potential conflict of interest.
